# Bacteriophages of Thermophilic ‘*Bacillus* Group’ Bacteria—A Review

**DOI:** 10.3390/microorganisms9071522

**Published:** 2021-07-16

**Authors:** Beata Łubkowska, Joanna Jeżewska-Frąckowiak, Ireneusz Sobolewski, Piotr M. Skowron

**Affiliations:** 1 Department of Molecular Biotechnology, Faculty of Chemistry, University of Gdansk, Wita Stwosza 63, 80-308 Gdansk, Poland; j.jezewska-frackowiak@ug.edu.pl (J.J.-F.); ireneusz.sobolewski@ug.edu.pl (I.S.); piotr.skowron@ug.edu.pl (P.M.S.); 2 The High School of Health in Gdansk, Pelplinska 7, 80-335 Gdansk, Poland

**Keywords:** bacteriophage, *Bacillus*, *Bacillus stearothermophilus*, *Geobacillus*, *Geobacillus stearothermophilus*, thermophile, bionanotechnology, probiotics, thermostable enzymes, TP-84

## Abstract

Bacteriophages of thermophiles are of increasing interest owing to their important roles in many biogeochemical, ecological processes and in biotechnology applications, including emerging bionanotechnology. However, due to lack of in-depth investigation, they are underrepresented in the known prokaryotic virosphere. Therefore, there is a considerable potential for the discovery of novel bacteriophage-host systems in various environments: marine and terrestrial hot springs, compost piles, soil, industrial hot waters, among others. This review aims at providing a reference compendium of thermophages characterized thus far, which infect the species of thermophilic ‘*Bacillus* group’ bacteria, mostly from *Geobacillus* sp. We have listed 56 thermophages, out of which the majority belong to the *Siphoviridae* family, others belong to the *Myoviridae* and *Podoviridae* families and, apparently, a few belong to the *Sphaerolipoviridae, Tectiviridae* or *Corticoviridae* families. All of their genomes are composed of dsDNA, either linear, circular or circularly permuted. Fourteen genomes have been sequenced; their sizes vary greatly from 35,055 bp to an exceptionally large genome of 160,590 bp. We have also included our unpublished data on TP-84, which infects *Geobacillus stearothermophilus* (*G. stearothermophilus*). Since the TP-84 genome sequence shows essentially no similarity to any previously characterized bacteriophage, we have defined TP-84 as a new species in the newly proposed genus *Tp84virus* within the *Siphoviridae* family. The information summary presented here may be helpful in comparative deciphering of the molecular basis of the thermophages’ biology, biotechnology and in analyzing the environmental aspects of the thermophages’ effect on the thermophile community.

## 1. Introduction

Bacterial viruses (bacteriophages or phages) are the most abundant, genetically and evolutionary diversified biological entities in the biosphere [1]. They play key roles in the regulation and evolution of bacterial communities in essentially all ecosystems. They are found in all environments, including extreme ones, such as those associated with: very low or high temperatures, hot/cold deserts, hypersaline systems, low or high pH, such as soda lakes, high hydrodynamic pressures or containing poisonous elements or compounds, among others.

Here we focus on a narrow segment of such bacteriophages, meeting two criteria: (i) those living in a moderately high temperature segment of approximately 45–70 °C. Ecosystems providing such a condition are diverse and include: terrestrial hot springs, deep-sea hydrothermal vents areas, shallow ocean waters above geothermally active beds, human generated hot waters, hot deserts, compost piles, greenhouse soils, silage, rotting straw, river sludge, stable manure, digested sewage sludge, among others. These contain thermophilic bacterial communities and specific viruses that infect them (thermophilic bacteriophages or thermophages) [2,3] and (ii) thermophages infecting a diverse ‘*Bacillus* group’ bacteria. A number of moderately thermophilic, spore-forming, aerobic bacteria with a growth range of 45–70 °C were classified into the genera *Aneurinibacillus*, *Alicyclobacillus*, *Sulfobacillus*, *Brevibacillus*, *Thermoactinomyces* and *Thermobacillus* [4,5,6,7]. However, genomes and proteomes have revealed that the majority of these thermophiles belong to the *Bacillus* sp. (*B*.) genetic groups 1 and 5 [8,9]. These findings further led to a more in-depth evaluation of thermophiles from group 5. In its course, it was determined, on the basis of high 16S rRNA sequence similarity (98.5–99.2%), that they form a phenotypically and phylogenetically coherent group of thermophilic bacilli. Thus, in 2001, the thermophiles belonging to *Bacillus* genetic group 5 were reclassified to be included into *Geobacillus* (*G.*) gen. nov. with the well-known *B. stearothermophilus* being assigned as the type strain *G. stearothermophilus* [9,10]. The *Geobacillus* name means earth or soil *Bacillus* and includes moderately thermophilic, aerobic or facultatively anaerobic, neutrophilic, motile, spore-forming rods [10]. The genus may also include some non-spore-forming bacteria, in which sporulation-related genes were inactivated by genome-integrated probacteriophage-like DNA segments [11]. The increasing number of discoveries of many novel thermophages, including those infecting the ‘*Bacillus* group’, are providing a more complete understanding of thermophiles’ biology, the mechanisms of biochemical adaptations needed for the life in high temperatures and the thermophilic host-bacteriophage evolution. From this perspective, especially interesting is the characterization of microbial and viral thermophilic communities in deep-sea hydrothermal vents fields, as this leads to deciphering the origin of ecosystems on the sea floor, the subsurface biosphere, the driving forces of evolution and the origin of life on the Earth [12,13]. Furthermore, it has been found that a horizontal gene transfer between thermophilic and mesophilic *Bacillus* species and bacteriophages takes place. This results in genetic mosaicism, as exemplified by the D6E thermophage, possibly leading to the contribution of substantial amounts of foreign DNAs by mobile DNA elements such as bacteriophages to entire microbial communities, including remote ones, such as the deep-sea vent community [14]. From a practical perspective, thermophilic *Bacillus* species are dominant bacterial workhorses in industrial fermentation processes, thus, these bacteria and their bacteriophages provide a novel source of genetic material and enzymes with great potential for application in biotechnology industry and science. Thus we provide an overview of the bacteriophages that infect thermophilic *Bacillus* sp. (many reclassified later as *Geobacillus* sp.), *Geobacillus* sp. and other related hosts that have been isolated from hot ecosystems around the world.

## 2. Environment Relation

Thermophilic bacilli, including *Geobacillus*, grow in essentially all environments, except those with ultra-harsh conditions, such as temperatures near or above boiling and extreme pH, among others. As mentioned above, geobacilli can be found in temperatures ranging from those typical for mesophiles (20–45 °C) up to those characteristic for moderate thermophiles (approximately 45–70 °C) in substantial numbers in ecological niches: geothermal areas, terrestrial hot springs, deep-sea hydrothermal vents areas, shallow ocean waters above geothermally active beds, human generated hot waters, hot deserts, compost piles, greenhouse soils, silage, rotting straw, river sludge, stable manure, digested sewage sludge, regardless of geographic location [15,16]. Especially rich sources of thermophilic *Geobacillus* bacteria, infected with bacteriophages, are present in humid organic matter, such as active compost piles and greenhouse soils due to active microbial metabolism and increased temperature, not reaching extreme values [2,17]. The *G. kaustophilus*-infecting thermophage GBK2 was found in a backyard compost pile after enrichment in liquid culture with *G. kaustophilus* (ATCC 8005) at 55 °C [18]. The minimum detectable growth temperature for *Geobacillus* isolates was described to be 45 °C [4], although in our hands, laboratory conditions allowed for a slow growth of. *G. stearothermophilus* strain 10 also in the mesophilic range. As typical soils rarely reach temperatures above 45 °C, this seems to be an evolutionary adaptation, allowing *Geobacillus* to grow extremely slowly, until more environmentally favorable growth temperatures occur. On the other hand, the temperatures in active organic matter easily exceed 45 °C. Very diverse conditions are present in hot springs: their temperatures range from 40 °C to 98 °C, and the bacteria capable of growing there, including geobacilli, are classified either as moderate thermophiles (40–71 °C) or hyperthermophiles (72–98 °C) [19]. Furthermore, the hot springs’ pH values vary widely, depending on hot water dissolving components of various minerals—from pH 1 to 9—and are defined in three functional categories [20]: acidic (pH 1–5), approximately neutral (pH 6–7.5) and alkaline (pH ˃ 7.5). This translates to a high diversity of ‘*Bacillus* group’ hosts and their specific bacteriophages, which are able to infect most of the major taxonomic groups of *Bacillus* thermophiles [21], regardless of the type of ecosystem. For example, two thermophages—*Bacillus* virus BVW1 and *Geobacillus* virus GVE1—were isolated from deep-sea hydrothermal fields in the east Pacific [22]. Thermostability assays showed that GVE1, whose host is *Geobacillus* sp. E26323, isolated from the same location, was most stable at 60 °C [23]. During the cultivation of *Geobacillus* sp. strain E263, many uncharacterized bacteriophage plaques were observed, indicating the presence of a rich host-bacteriophage community. Another deep-sea hydrothermal fields thermophage GVE2 was found in geobacilli cultured at 65 °C [14,24]. Additionally, a *Geobacillus* thermophage D6E was characterized in deep-sea hydrothermal vent communities, where viruses play very important roles [14]. Yellowstone National Park is one of the best studied sites regarding the thermophages that infect the species of *Geobacillus Thermus* as well as *Archea*, among others. This is due to the fact that Yellowstone has one of the highest concentrations of hot springs in the world, which vary greatly in terms of temperature, pH and minerals content [10]. Bacteriophage GBSV1, infecting *Geobacillus* sp. 6k51 was isolated from a shallow (100 m depth) off-shore hot spring [25]. Similarly, the bacteriophage ϕOH2, which infects *G. kaustophilus* NBRC 102445(T), was isolated from the sediment of a hot spring [26] and bacteriophage AP45 and its host strain *Aeribacillus* sp. CEMTC656 were isolated from the Valley of Geysers, Kamchatka, Russia [27].

**Figure 1:**
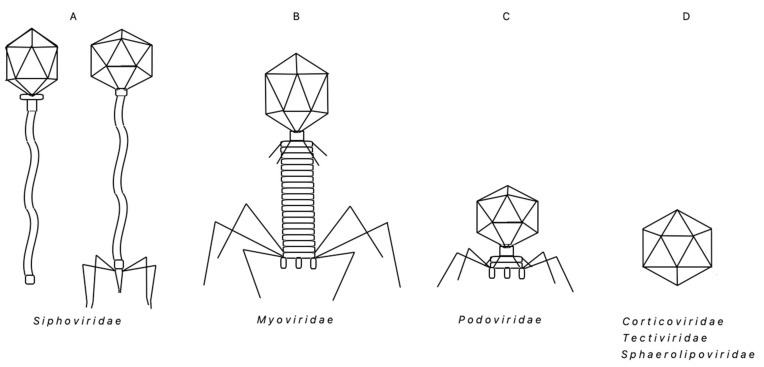
Schematic representation of morphological/structural forms of the ‘*Bacillus* group’ thermophages. (**A**). *Siphoviridae*; (**B**). *Myoviridae*; (**C**). *Podoviridae*; (**D**). *Sphaerolipoviridae*, *Tectiviridae*, *Corticoviridae*.

Regardless of the environmental diversity, the morphologies and compositions of the ‘*Bacillus* group’ thermophilic bacteriophages are less diverse than that of the overall community of thermophages. In general, the thermophilic bacteriophages that have been found in all taxonomic groups of *Bacteria* and *Archaea*, exhibit diverse morphologies (e.g., tailed capsids, tail-less capsids, filamentous, lipid-containing), unique structures (e.g., very long-tailed *siphoviruses*), and extend through five taxonomic families: *Myoviridae, Siphoviridae, Inoviridae, Tectiviridae* and *Sphaerolipoviridae* [3]. Interestingly, in the diverse ‘*Bacillus* group’ bacteria, the bacteriophages found thus far belong to a limited number of families: vastly dominating *Siphoviridae* (head–tail bacteriophages with non-contractile tails), *Myoviridae* (head–tail bacteriophages with contractile tails), *Podoviridae* (very short tail) and, possibly scarce, *Sphaerolipoviridae, Tectiviridae or Corticoviridae* members (icosahedral, with a lipid membrane) (Figure 1). All of them contain dsDNA genome, either linear or circular, or circularly permitted. On the whole, the ubiquity of thermophages and their impact on various trophic levels seems to have a powerful direct influence on diverse microbial communities that play a crucial role in biogeochemical cycles. All this, in turn, plays a role in altering those cycles [27,28,29]. As a result, thermophages can affect/stimulate the modifications of those cycles.

## 3. ‘*Bacillus* Group’ Bacteriophages—Chronological Review

The thermophilic *‘Bacillus* group’ bacteriophages, reviewed here, comprise 56 species, described in varying detail in original publications (Table 1). Although there have certainly been more such bacteriophages detected, they have not been sufficiently analyzed or have been just briefly mentioned in publications. They infect various *Geobacillus* (previously *Bacillus*) sp., ‘*B. stearothermophilus’*, *B. acidocaldarius*, *B. caldotenax*, *B. caldolyticus*, *G. kaustophilus*, *G. thermoglucosidasius*, *G. icigianus*, *B. pseudoalcaliphilus*, *B. bogoriensis*, *B. pseudofirmus*, *B. thermocatenulatus*, *B. cohnii*, undefined *Bacillus* sp. and *Aeribacillus* sp. As older species determinations are not always precise, we tend to retain the original author’s nomenclature to avoid confusion and for the reader’s convenience, when reading original publications. Some of these thermophages were discovered and analyzed as long ago as the 1950s, 1960s and 1970s, using the techniques available at the time. The data thus obtained is often incomplete or contains errors (such as the nearly 2-fold underestimation of TP-84’s genome size) especially at the molecular level. Apparently, the earliest thorough/reliable report is that of the thermophage TP-84, discovered in 1952 and preliminarily characterized in the 1960s and 1970s [30]. Much later, its genome was sequenced, analyzed and its proteomics data was established by our group in 2018 [2] (Table 1). Below, we chronologically review the major features of each thermophage and its interactions with its corresponding host. Interestingly, all 56 bacteriophages contain dsDNA genomes. The hosts and bacteriophages were cultivated typically on rich, ‘nutrient broth’-type media at approximately pH 7, unless otherwise noted.

### 3.1. ‘Thermophilic Lytic Principle’ (Family/Genus Not Determined (ND), Host Putative G. stearothermophilus)

This is apparently the earliest discovered and reported thermophilic bacteriophage, with no assigned name, just mentioned as thermophilic lytic principle [31]. Its host, T60 bacteria was described as medium length, Gram-positive rods, isolated from milk, forming spores and growing between 20 °C and 60 °C, with the optimum at 45–52 °C, which suggests that the host belongs to *G. stearothermophilus*. The thermophilic lytic principle was capable of inoculating between series of cultures and was optimally lysing the host’s cells at 52–55 °C; the lysis capability declining sharply at temperatures of 60 °C and with no lysis occurring at 62 °C [31].

### 3.2. ‘Thermophilic Bacteriophage’ (1) (Family/Genus ND, Host Thermophilic Bacillus sp.)

The bacteriophage, isolated by Adant in 1928 [32], had similar properties to the thermophilic lytic principle [30]. It was optimally active at 52 °C and was destroyed in 30 min. at 75 °C.

### 3.3. ’Thermophilic Bacteriophage’ (2) (Family/Genus ND, Host ‘Thermophilic Bacterium No. 10′)

Scarce data was published concerning thermophilic bacteriophage [33]. It was found to infect a thermophilic bacterium no. 10, aerobic, Gram-positive, isolated from thermal springs in Yellowstone National Park, as being or closely resembling *B. stearothermophilus* [34]. The bacteria exhibited temperature growth range of 35–75 °C and the bacteriophage was propagated with stimulating adsorption Ca^2+^ ions at 50–70 °C, with optimum at 65 °C, and a latent period of 80–90 min. The bacteriophage produced a halo around clear plaques, suggesting the existence of a diffusing envelope depolymerase. It exhibited substantial thermostability. At 2 h incubation at 95 °C in its growth media, significant phage titer remained, whereas at 100 °C after 30 min, some infective particles were still detectable. Transmission electron microscopy (TEM) images showing the bacteriophage morphology suggest that it belongs to the *Siphoviridae* family [33,35].

### 3.4. ‘Thermophilic Bacteriophage’ (3) (Family/Genus ND, Host Thermophilic Bacillus sp.)

One of the first isolated thermophilic bacteriophages included an undefined species of very high thermostability. As a host served gram-positive bacillus, spore-forming, strictly aerobic, capable of growth at over 70 °C, isolated from a compost pile in Tokyo, Japan [36]. The bacteriophage was capable of lysing a host’s cells at between 55 °C and 70 °C, optimally at 65 °C, with a burst size of 205–220. Remarkably, that bacteriophage exhibited a very high thermostability. Suspended in its cultivation media, it was capable of surviving over 120 min at 100 °C with approximately a 1000-fold decrease in a titer. The bacteriophage TEM analysis has shown spherical particles of approximately 20 nm diameter with a tendency to aggregate. Aside from that, the results of the coding nucleic acid determination are questionable. The author claims, as based on chemical analysis, that the bacteriophage is composed of 74% nucleic acids, both DNA—42%, and RNA—32% [37,38].

### 3.5. TP-84 (Siphoviridae Family, Tp84virus Genus, Host B. stearothermophilus)

Early studies determined a number of microbiological and physical properties of bacteriophage TP-84. This was discovered in 1952 in greenhouse soil, infecting *B. stearothermophilus* strain 2184 (according to current classification—*G. stearothermophilus*) [30]. TP-84 exhibits high host specificity. Out of +24 related strains of *B. stearothermophilus*, two unclassified thermophilic *Bacillus* strains, T-27 and 194, also supported TP-84 infection as well as *G. stearothermophilus* strain 10 and 4 supporting TP-84 growth. The most effective host was *G. stearothermophilus* strain 10, thus it was further used for more detailed studies [39]. Here, we evaluated more *G. stearothermophilus* hosts that have been used by others for recombinant DNA studies in these bacteria, including: NUB3625 9A5 and NUB R [40]. These hosts supported TP-84 growth but much less effectively than *G. stearothermophilus* 10. The cultivation conditions, optimal for TP-84, included a rich media of pH 6.5, supplemented with fructose or glucose as a sporulation suppressor and essential Ca^2+^ and Mg^2+^ ions. TP-84 can reach a high titer of app. 10^12^. However, omitting the sugar addition decreased the titer approximately 1000-fold [39], while omitting divalent cations had a dramatic effect on final TP-84 yields, causing an approximate 200-fold decrease in titer [41]. The temperature growth range of 43–76 °C (optimal 55–60 °C) matched that of the hosts growth range. The latent period was 22–24 min. Clear plaques were surrounded by a turbid ‘halo’ ([41] Lubkowska et al., unpublished data), indicating diffusion of an enzyme, possibly glycosylase-depolymerase, degrading the bacterial envelopes. The bacteriophage has a hexagonal head, 53 nm in diameter with a 131 nm long 3–5 nm wide tail. It has a Svedberg coefficient of 436 and bands in CsCl at a density of 1.508 g/cm^3^ (pH 8.5). The sedimentation and diffusion data allowed for calculation of the TP-84 particle molecular weight of approximately 50 MDa [39]. In our previous work, we characterized and sequenced the genome, and established the proteomics data on TP-84 [2]. TP-84 contains 47.7 kb dsDNA, 42% G+C genome. The bioinformatics analysis revealed the presence of 81 coding sequences (CDSs), coding for polypeptides of 4 kDa or larger. Interestingly, all CDSs are oriented in the same direction, pointing to a dominant transcription direction of one DNA strand only. Based on homology analysis, a hypothetical function could be assigned to 31 CDSs and a total of 33 biosyntheses of proteins was confirmed experimentally [2]. To further confirm the genome bioinformatics data, we analyzed: (i) purified TP-84 bacteriophage particles, subjected to SDS-PAGE, resolving individual proteins and (ii) purified TP-84 proteins from TP-84/*G. stearothermophilus* lysates, followed by LC-MS analysis (to be published elsewhere). Nineteen new TP-84-encoded proteins were found (Table 1) with at least two peptides sequence coverage. Those include: (i) proteins involved in DNA replication/packaging—TP84_01 (putative terminase, small subunit), TP84_02 (putative terminase, large subunit), TP84_59, replicative DNA helicase and TP84_58, replicative helicase inhibitor; (ii) TP84_27—holin, involved in cell membrane disintegration during host lysis; (iii) TP84_61—HNH homing endonuclease, which may be involved in recombination processes; (iv) TP84_26—glycosylase, possibly involved in disintegration of a very thick host’s envelope. This is the largest protein of TP-84 (112 kDa) and, interestingly, it shows homology to a number of proteins from *Bacillus* and *Geobacillus* species, and one *Geobacillus* bacteriophage GBK2 protein (GenBank YP_009010491). No RNA or DNA polymerase-coding genes were found on the TP-84 genome, thus the bacteriophage relies on the host’s transcription and replication enzymes. The TP-84 genome has a ‘condensed’ genome with CDSs, typically spaced by several-to-tens of bp only and often overlapping. The genome contains five putative promoter-like sequences resembling the host promoter consensus sequence with allowed 2-bp mismatches. Furthermore, ten putative rho-independent terminators were found. The TP-84 genome sequence shows essentially no similarity to any previously characterized bacteriophage, thus, TP-84 was established as a holotype of a new genus *Tp84virus* within the *Siphoviridae* family [2].

### 3.6. φμ-4 (Family/Genus Not Determined, Host B. stearothermophilus)

Bacteriophage φμ-4 was isolated from lysogenic *B. stearothermophilus* NU strain 4, and was propagated in *B. stearothermophilus* 10. It was cultivated at 50–65 °C with required Ca^2+^ ions. The latent period at 65 °C was 35 min with a burst size of 175. TEM images have shown the bacteriophage particles size of 88–100 nm. The particles shape was ‘roughly spherical’ [42].

### 3.7. TP-1 (Putative Siphoviridae Family, Host B. stearothermophilus)

TP-1 bacteriophage was isolated as a mitomycin- or UV-induced lysogenic *B. stearothermophilus* 1503-4R, growing in the narrow temperature range of 50–65 °C, with optimum at 55 °C. Ca^2+^ ions enhanced the bacteriophage propagation. TP-1 formed turbid plaques when titrated on *B. stearothermophilus* 4S. Furthermore, a clear-plaque mutant of TP-1C was found. Lysogen induction was rapid, causing a culture lysis within 45–60 min. Spontaneous induction was detected at a rate of 1 particle/app. 3 × 10^6^ cells. Morphologically, TP-1 has a head of 65 nm and a long, flexible tail of 240 nm and 12 nm wide, which suggests that TP-1 belongs to the *Siphoviridae* family. The bacteriophage genome is composed of 42% G+C, dsDNA, with estimated molecular weight of 1.21 × 10^7^ [43,44], which corresponds to approximately 18,516 bp. One of the authors [45] isolated a number of thermophilic bacteriophages, exemplified by TP-42, TP-56 and TP-68, but no characterization was provided.

### 3.8. ST1 (Siphoviridae Family, Genus ND, Host B. stearothermophilus)

Bacteriophage ST1 was propagated at 60 °C in *B. stearothermophilus* S13, forming clear plaques. TEM micrographs showed the head of ST1 as a regular icosahedral form of 50 nm in diameter, a tail of 100 nm length and 2 nm wide, ended with a terminal plate. The genetic material was dsDNA, containing 43% G+C [46].

### 3.9. Tφ3 (Tphi3) (Siphoviridae Family, Genus ND, Host G. stearothermophilus)

Tphi3 bacteriophage was isolated from a soil sample and infects the thermophilic *B. stearothermophilus* ATCC 8005. Along with Tphi3, there were two more bacteriophages isolated, apparently infecting *B. stearothermophilus*, not further characterized. Tphi3 is highly host-specific, as it does not infect *B. stearothermophilus* ATCC 7953, 7954, 10,149, 12,016. Optimal growth occurs at 60 °C with a latent period of 18 min and burst size of approximately 200. The Tphi3 exhibits high thermostability, up to 75 °C with a half-life of 12 min. Prolonged plates incubation resulted in a formation of a turbid ‘halo’ around clear plaques, suggesting a secretion of an enzyme, depolymerase, degrading the bacterial envelope. TEM imaging of Tphi3 shows that the bacteriophage has a 57 nm long regular icosahedron head. Edges of the head are 29 nm long, the tail is 125 nm long and 10 nm wide. The tail contains a stacked-ring structure. There are about 30 cross-strations that are spaced at 3.9-nm intervals along the tail and TEM images also suggest that the tail end contains small fibers. The buoyant density of the bacteriophage in a cesium chloride density gradient was 1.526 g/mL. Ca^2+^ ions were required for efficient adsorption and/or growth [47]. Further reported Tphi3 DNA studies, indicated that its average genome length was 12.1 mm, which corresponds to app. 23.2 MDa or app. 35,700 bp with G+C content of 40.2% [48].

### 3.10. GH5, GH8 (Family/Genus ND, Host B. stearothermophilus) 

A brief report by Humbert and Fields (1972) showed two bacteriophages, GH5 and GH8, with distinct features, both infecting *B. stearothermophilus* NCA1518 [49]. Both share the same plaque-forming temperature range of 42.5–67 °C. Bacteriophage ND8 was examined under TEM and exhibited an icosahedral head with a diameter of approximately 100 nm, a long tail of 330 nm and approximately 10 nm wide, thus, apparently belonging to the *Siphoviridae* family. The latent periods were 47 min (GH5) or 35 min (GH8), burst size 51 (GH5) or 72 (GH8), buoyant density 1.473 (GH5) or 1.506 (GH8), sensitivity to osmotic shock from 2 M sucrose (both), sensitivity to osmotic shock from 4 M NaCl (GH5 no, GH8 yes), extrapolated 1 min half-life 88 °C (GH5) or 76 °C (GH8), maximum half-life at pH 9.0 (GH5) or 7.0 (GH8) [49].

### 3.11. PhB1 (Siphoviridae Family, Genus ND, Host Thermophilic Bacillus sp.)

PhB1 was isolated from a farm soil sample, using a soil-isolated bacterium as a host, described by the authors as facultatively thermophilic *Bacillus* sp. strain B1, Gram-positive rods, aerobic, spore-forming, motile with temperature growth range approximately 30–60 °C and optimum between 45–50 °C. The bacteriophage was cultivated on the hosts, growing at 55 °C. Small plaques were obtained with PhB1 and had a clear center with a diameter less than one millimeter, surrounded by a turbid halo [50], which suggests the presence of secreted envelope depolymerases. It was determined that Ca^2+^ ions and Mg^2+^ ions were stabilizing the bacteriophage particles against heat inactivation. TEM showed a long-tailed particle with a probable octahedral head and small spikes at the tail tip.

### 3.12. D5–D8 (Family/Genus ND, Host B. stearothermophilus) 

A report by Reanney and Marsch (1973) presented 4 bacteriophages: D5, D6, D7, D8 infecting thermophilic *B. stearothermophilus* NRS T91 and ATCC7953. The hosts were grown on rich media supplemented with glucose and Ca^2+^ ions. Remarkably, the plaque-forming range for the bacteriophages extended down to the mesophilic region of 30–37 °C, with an optimum at 45 °C and reaching the upper limit at 55 °C. Bacteriophage proliferation in soil was greatest at 45 °C, while at 55 °C this was much less intense. The authors raised a hypothesis concerning the ecology of bacteriophages infecting the thermophilic soil organism *B. stearothermophilus*. The authors suggested that, in soil, these bacteria and their bacteriophages proliferate optimally at mesophilic rather than thermophilic temperatures. They proposed that, in the field, the vast majority of the metabolizing population of *B. stearothermophilus* are mesophilic in their biochemistry and that the ‘thermophilic’ strains, maintained in laboratories, represent a selection obtained by the isolation procedure of a minority of atypical, thermophilic cells [51].

### 3.13. φNS11 (PhiNS11) (Putative Sphaerolipoviridae, Tectiviridae or Corticoviridae Family, Host Acidophilic-Thermophilic B. acidocaldarius)

φNS11 is a ‘double’ extremophilic bacteriophage, as it infects both acidophilic and thermophilic bacterium, *A. acidocaldarius* strain TA6, growing in the Beppu hot springs in Japan. The bacteriophage was propagated in a pH range of 2 to 5 and exhibited optimum growth at 60 °C and pH 3.5. Above pH 6, it became unstable. φNS11 has a polyhedral shape, is tail-less and has a lipid-containing capsid with a diameter of 60–70 nm and spike-like structures. SDS-PAGE showed that φNS11 contains only six proteins. The genome was determined to be composed of dsDNA. The buoyant density of the bacteriophage was relatively low, 1.27 g/cm^3^, similar to that of the lipid-containing phages PM2, PR4, φ6. Furthermore, it was highly sensitive to organic solvents. The fatty acid composition of the bacteriophage was slightly different from that of the host bacterium. However, o-cyclohexyl fatty acids typical of *B. acidocaldarius*, were also found in φNS11 as its main components [52]. These features point to classification of φNS11 as belonging to the *Sphaerolipoviridae, Tectiviridae or Corticoviridae* families.

### 3.14. JS001-JS027 Series (Section 3.14, Section 3.15, Section 3.16, Section 3.17, Section 3.18, Section 3.19, Section 3.20, Section 3.21, Section 3.22, Section 3.23, Section 3.24, Section 3.25, Section 3.26, Section 3.27, Section 3.28, Section 3.29, Section 3.30, Section 3.31, Section 3.32, Section 3.33, Section 3.34, Section 3.35, Section 3.36 and Section 3.37 Below)

Sharp et al. (1986) have discovered and partially characterized 24 thermophilic bacteriophages, designated JS001-JS027, isolated from diversified sources: compost piles, soil, mud, sewage and river sludges, stable manure, silage and rotting straw, capable of infection of most of the major taxonomic groups of *Bacillus* thermophiles [21]. Various species/strains were used for the bacteriophages detection and propagation, including: *‘B. caldotenax’* DSM 406, DSM 411, *‘B. caldolyticus’* DSM 405, the *Bacillus* thermophile RS 93, *B. stearothermophilus* NCA 1503, NW 10, EP 240, EP 262, EP 136, DSM 2334, ATCC 12016 [53,54], streptomycin-resistant strains of: *B. stearothermophilus* NCA 1503, *Bacillus* thermophiles RS 239, RS 240, RS 241 and RS 242 [55]. The bacteriophages were uniformly detected/propagated in rich media of pH app. 7, and supplemented with Ca^2+^ ions, at 55 °C. They exhibited significant differences in capsid morphology, comprising 4 groups: (i) comparatively short stubby tail and no evidence of a tail plate; (ii) icosahedral heads of approximately 40–65 nm in diameter with tails between 120 nm and 135 nm long and no evidence of a tail plate; (iii) icosahedral head of 85–90 nm in diameter and a long (400 nm) flexible tail with helical symmetry; (iv) cylindrical heads with rounded ends, with tails varying between 115 nm and 120 nm long. All the genomes were determined as dsDNA. A number of isolates were lost when chloroform was used to sterilize them, indicating vital lipids content or denaturation-sensitive capsid protein(s). Table 2 contains selected data, concerning JS001–JS027.

### 3.15. JS001 (Family/Genus ND, Hosts Thermophilic Bacillus sp.)

JS001 was obtained by induction of *B. stearothermophilus* NCA 1503 and propagated on *B. caldotenax.* Other susceptible hosts included: *B. stearothermophilus*: ATCC 12016, RS 93, EP 262; *Bacillus* thermophile: RS 15, RS 108, RS 125 [21].

### 3.16. JS004 (Putative Podoviridae Family, Hosts Thermophilic Bacillus sp.)

JS004 was isolated from a silage, propagated on *Bacillus* thermophile RS 239, yielding small 0.5–1 mm circular, hazy plaques. High thermal stability was observed up to 60 °C, then gradually declining, but still substantial at 70 °C. Other susceptible hosts included: *B. stearothermophilus* RS 93, *Bacillus* thermophile RS 241. The bacteriophage belongs to the group 1: it possesses an icosahedral head 55–60 nm long, a short cylindrical 15 nm stubby tail with collar and no evidence of a base plate, which suggests that JS004 belongs to the *Podoviridae* family [21].

### 3.17. JS005 (Family/Genu ND, Hosts Thermophilic Bacillus sp.)

JS005 was isolated from rotting straw, propagated on *Bacillus* thermophile RS 239 and yielding small 0.5–1 mm circular, slightly hazy plaques. The only other susceptible host was *Bacillus* thermophile RS 241 [21].

### 3.18. JS006 (Family/Genus ND, Hosts Thermophilic Bacillus sp.)

JS006 was isolated from compost, propagated on *Bacillus* thermophile RS 239 and yielding small 0.5–0.75 mm circular, clear plaques. High thermal stability was observed up to 60 °C, then gradually declining, but still substantial at 70 °C. Other susceptible hosts included: *B. stearothermophilus*: RS 93, NW 4S, EP 262, *Bacillus* thermophile RS 242 [21].

### 3.19. JS007 (Family/Genus ND, Hosts Thermophilic Bacillus sp.)

JS007 was isolated from silage, propagated on *Bacillus* thermophile RS 240, and yielding small 0.5–0.75 mm circular, clear plaques. High thermal stability was observed up to 60 °C, then gradually declining, but still substantial at 70 °C. Other susceptible hosts included: *B. stearothermophilus* RS 93, *and Bacillus* thermophile RS 239, RS 240, RS 241. The bacteriophage belongs to the group 3: it possesses an icosahedral head 80–85 nm, an exceptionally long flexible tail of 400–420 nm with cross-strations and a fine fiber at tip, which suggests that JS007 belongs to the *Siphoviridae* family [21].

### 3.20. JS008 (Family/Genus ND, Hosts Thermophilic Bacillus sp.)

JS008 was isolated from rotting straw, propagated on *Bacillus* thermophile RS 241, yielding 1–3.5 mm circular, hazy plaques. Another susceptible host was *Bacillus* thermophile RS 239 [21]. 

### 3.21. JS009 (Family/Genus ND, Hosts Thermophilic Bacillus sp.)

JS009 was isolated from stable manure, propagated on *Bacillus* thermophile RS 242 and yielding small 0.5 mm circular plaques with a hazy center. Other susceptible hosts included*: B. stearothermophilus* RS 93, *Bacillus* thermophile RS 241 [21].

### 3.22. JS010 (Family/Genus ND, Hosts Thermophilic Bacillus sp.)

JS010 was isolated from compost, propagated on *Bacillus* thermophile RS 242 and yielding 3 mm, irregular, hazy plaques with clear areas at the edge. Other susceptible hosts included: *B. stearothermophilus* RS93, *Bacillus* thermophile RS 241 [21].

### 3.23. JS011 (Family/Genus ND, Hosts Thermophilic Bacillus sp.)

J7S011 was isolated from silage, propagated on *Bacillus* thermophile RS 239 and yielding 3 mm, irregular, hazy plaques with clear areas at the edge [21].

### 3.24. JS012 (Family/Genus ND, Hosts Thermophilic Bacillus sp.)

JS012 was isolated from compost, propagated on *Bacillus* thermophile RS 239 and yielding 0.5–2 mm, irregular, clear plaques [21].

### 3.25. JS013 (Family/Genus ND, Hosts Thermophilic Bacillus sp.)

JS013 was isolated from soil, propagated on *B. stearothermophilus* NCA 1503 and yielding 0.75–1 mm, circular, hazy plaques [21].

### 3.26. JS014 (Putative Siphoviridae, Hosts Thermophilic Bacillus sp.)

JS014 was isolated from rotting straw, propagated on *B. stearothermophilus* NCA 1503 and yielding small 0.5–1 mm circular, slightly hazy plaques. High thermal stability was observed up to 60 °C, then gradually declining, but still substantial at 70 °C. Another susceptible host was *B. stearothermophilus* NW 10. The bacteriophage belongs to the group 3: it possesses icosahedral head 85–90 nm, an exceptionally long flexible tail of 400 nm with cross-strations, which suggests that JS007 belongs to the *Siphoviridae* family [21].

### 3.27. JS015 (Family/Genus ND, Hosts Thermophilic Bacillus sp.)

JS015 was isolated from compost, propagated on *B. stearothermophilus* NCA 1503 and yielding 0.5–2 mm, circular, slightly hazy plaques. Another susceptible host was *B. stearothermophilus* NW 10 [21].

### 3.28. JS017 (Putative Myoviridae Family, Hosts Thermophilic Bacillus sp.)

JS017 was isolated from compost, propagated on *B. caldotenax* and yielding small 1–1.5 mm circular, turbid/hazy center plaques. High thermal stability was observed up to 60 °C, then gradually declining, but still substantial at 70 °C. Other susceptible hosts included: *B. caldotenax* DSM 406, *B. caldovelox* DSM 411, *B. caldolyticus* DSM 405, *B. stearothermophilus*: ATCC 12016, NW 10, RS 93, EP 262, EP 240, ATCC 8005, *B. thermocatenulatus* DSM 730. The JS017 belongs to the group 4: it possesses a cylindrical head with round ends 80 × 40 nm, a rigid tail of 115 nm with cross-strations and triangular base plate, which suggests that JS017 belongs to the *Myoviridae* family. Interestingly, a 1–5% portion of the bacteriophage population had heads of twice the normal length and/or less frequently longer tails [21]. 

### 3.29. JS018 (Putative Myoviridae Family, Hosts Thermophilic B. caldotenax)

JS018 was isolated from rotting vegetation, propagated on B. *stearothermophilus* NCA 1503 and yielding 1 mm, circular, clear center plaques. Other susceptible hosts included: *B. caldotenax* DSM 406, *B. caldovelox* DSM 411; *B. caldolyticus* DSM 405*, B. stearothermophilus*: RS 93, EP 240 [21].

### 3.30. JS019 (Putative Myoviridae Family, Hosts Thermophilic B. caldotenax)

JS019 was isolated from rotting vegetation, propagated on *B. caldotenax* and yielding small 1–1.5 mm circular, clear center, hazy at edges plaques. High thermal stability was observed up to 60 °C, then gradually declining, but still substantial at 70 °C. The bacteriophage exhibited broad specificity. Other susceptible hosts included: *B. caldotenax* DSM 406, *B. caldovelox* DSM 411; *B. caldolyticus* DSM 405, *B. stearothermophilus*: RS 93, EP 240. The JS019 belongs to the group 2: it possesses an icosahedral head of 50 nm and rigid tail of 130, which suggests that JS019 belongs to the *Myoviridae* family [21]. 

### 3.31. JS020 (Family/Genus ND, Hosts Thermophilic B. caldotenax)

JS020 was isolated from rotting vegetation, propagated on *B. caldotenax* and yielding 1–2 mm, circular, clear center, hazy outer halo 4–8 mm plaques. This suggest that the bacteriophage produces very active depolymerase, degrading the host’s envelope. Other susceptible hosts included: *B. caldotenax* DSM 406, *B. caldovelox* DSM 411, *B. caldolyticus* DSM 405, *B. stearothermophilus*: RS 93, EP 240 [21]. 

### 3.32. JS021 (Family/Genus ND, Hosts Thermophilic B. caldotenax)

JS021 was isolated from rotting vegetation, propagated on *B. caldotenax* and yielding 2–3 mm, circular, clear centre, hazy outer halo plaques. This suggests that the bacteriophage produces an envelope depolymerase. Other susceptible hosts included: *B. caldotenax* DSM 406, *B. caldovelox* DSM 411; *B. caldolyticus* DSM 405, *B. stearothermophilus*: RS 93, EP 240, ATCC 12016 [21]. 

### 3.33. JS022 (Putative Myoviridae Family, Hosts Thermophilic B. caldotenax)

JS022 was isolated from compost, propagated on *B. caldotenax* and yielding 1–2 mm, circular, clear centre plaques. Other susceptible hosts included: *B. caldotenax* DSM 406, *B. caldovelox* DSM 411; *B. caldolyticus* DSM 405, *B. stearothermophilus*: RS 93, EP 240. The JS022 belongs to the group 2: it possesses an icosahedral head 60–65 nm long and a rigid tail of 130 nm, which suggests that JS022 belongs to the *Myoviridae* family [21].

### 3.34. JS023 (Family/Genus ND, Hosts Thermophilic B. caldotenax)

JS023 was isolated from compost, propagated on *B. caldotenax* and yielding 0.5–1.5 mm, circular, clear centre, hazy edge plaques. Other susceptible hosts included: *B. caldotenax* DSM 406, *B. caldovelox* DSM 411; *B. caldolyticus* DSM 405, *B. stearothermophilus*: RS 93, EP 136 [21].

### 3.35. JS024 (Putative Siphoviridae or Myoviridae Family, Hosts Thermophilic B. caldotenax)

JS024 was isolated from compost, propagated on *B. caldotenax* and yielding 0.5–1.5 mm, circular, clear center, hazy edge plaques with a less hazy outer edge. Other susceptible hosts included: *B. caldotenax* DSM 406, *B. caldovelox* DSM 411; *B. caldolyticus* DSM 405, *B. stearothermophilus*: RS 93, EP 240, EP 136. The JS024 belongs to the group 2: it possesses an icosahedral head 60 nm long and a tail of limited flexibility 120 nm, which suggests that JS024 belongs to either the *Siphoviridae* or *Myoviridae* family [21].

### 3.36. JS025 (Putative Myoviridae Family, Hosts Thermophilic B. caldotenax)

JS025 was isolated from compost, propagated on *B. caldotenax* and yielding 1–1.5 mm, circular, hazy plaques. Other susceptible hosts included: *B. caldotenax* DSM 406, *B. caldovelox* DSM 411; *B. caldolyticus* DSM 405, *B. stearothermophilus*: RS 93, EP 240. The JS025 belongs to the group 2: it possesses an icosahedral head 45–50 nm long and a rigid tail of 120 nm, which suggests that JS025 belongs to the *Myoviridae* family [21].

### 3.37. JS026 (Putative Siphoviridae or Myoviridae Family, Hosts Thermophilic B. caldotenax)

JS026 was isolated from compost, propagated on *B. caldotenax* and yielding 1–1.5 mm, circular, hazy plaques. Other susceptible hosts included: *B. caldotenax* DSM 406, *B. caldovelox* DSM 411; *B. caldolyticus* DSM 405, *B. stearothermophilus*: RS 93, ATCC 12016. The JS026 belongs to the group 4: it possesses a cylindrical head with round ends 80 × 45–50 nm and a limited flexibility tail of 120 nm, which suggests that JS026 belongs to either the *Siphoviridae* or *Myoviridae* family [21].

### 3.38. JS027 (Putative Podoviridae Family, Hosts Thermophilic Bacillus sp.)

JS027 was isolated from compost, propagated on *Bacillus* thermophile RS241 and yielding 0.75 mm, circular, clear plaques. Another susceptible host is *Bacillus* thermophile RS239. The JS027 belongs to the group 1: it possesses an icosahedral head 55–60 nm long and a very short, stubby tail with no evidence of a tail plate, which suggests that JS027 belongs to the *Podoviridae* family [21].

### 3.39. W1 (BVW1) (Siphoviridae Family, Genus ND, Host Thermophilic Bacillus sp.)

W1 (BVW1) was isolated from a deep-sea hydrothermal field, located in the west Pacific (19_ 24′08″ N, 148_44′79″ E, depth of 5060 m), along with its host *Bacillus* sp. W13 (GenBank AY583457), which is aerobic, rod-shaped and spore-forming, growing within a temperature range of 45–85 °C with an optimum at 65–70 °C. The host range was narrow, as the W1 infects only one thermophilic strain out of 10 tested. The bacteriophage propagates most effectively at 60 °C. Morphologically, W1 has a hexagonal head of 70 nm in diameter and a very long tail of 300 nm and 15 nm in width. The bacteriophage genome is composed of dsDNA with an estimated size of 18 kb. As determined, using SDS-PAGE of purified virions, six major proteins were detected with estimated molecular weights: 32, 45, 50, 57, 60 and 70 kDa [22].

### 3.40. GVE1 (E1) (Siphoviridae Family, Genu NDs, Host Geobacillus sp.)

GVE1 was isolated from a deep-sea hydrothermal field, located in the east Pacific (12_42′29″ N, 104_02′01″ W, depth of 3083 m), along with its host *Geobacillus* sp. E26323 (GenBank DQ225186), which is aerobic, rod-shaped and spore-forming, growing in rich media of pH 7, within a temperature range of 45–85 °C and an optimum at 65–70 °C. The host range is narrow, as the GVE1 infects only one thermophilic strain out of 10 tested. The bacteriophage propagates most effectively at 60 °C. GVE1 possesses a large hexagonal head of 130 nm in diameter, a tail of 180 nm and 30 nm in width. The bacteriophage genome is composed of dsDNA with an estimated size of 41 kb. As determined on SDS-PAGE of purified virions, six major proteins were detected with estimated molecular weights: 34, 37, 43, 60, 66 and 100 kDa [22].

### 3.41. GVE2 (E2) (Siphoviridae Family, Genus ND, Hosts Geobacillus sp.)

Siphovirus GVE2 (also known as E2) is one of the very few deeply studied *Geobacillus* thermophages and was isolated from the same location as GVE1 [22]. The authors in the publications concerning GVE2, refer to their previous publication concerning the discovery of GVE1, thus, it is possible that the bacteriophages are identical or closely related.

GVE2 is a virulent *Siphoviridae* bacteriophage infecting deep-sea thermophilic *Geobacillus* sp. E263 (CGMCC1.7046), capable of growth at 45–80 °C, with optimum at 60–65 °C, which was selected for GVE2 propagation. The bacteriophage genome is a 40,863 bp, 44.8% G+C, linear dsDNA with 62 ORFs [22]. Its genome shows sequence similarity to the genomes of several *Geobacillus* sp. bacteriophages. Six GVE2 proteins were characterized in the laboratory. Transcriptomic analysis has shown that 74.2%, 46 of the ORFs, were transcribed and potentially expressed as proteins. The six structural proteins of purified GVE2 virions were also determined by mass spectrometry and, for one of those, novel portal protein VP411 protein, its coding gene was cloned/expressed in *Escherichia coli* (*E. coli*). The recombinant VP411 was characterised in details and confirmed by immune electron microscopy using gold-labelled secondary antibody, as located in the portal-neck region of the GVE2 [24]. Subsequently, the endolysin coding gene was cloned in *E. coli*, expressed and the protein purified and characterised [56]. Further, the holin-endolysin system was studied and it was shown that GFP–endolysin fusion aggregated in GVE2-infected *Geobacillus* sp. and revealed that the endolysin and holin interacted directly as well, as with the host *Geobacillus* protein ABC transporter system, which apparently participates in the regulation of the lysis process [57].

### 3.42. GBSV1 (Myoviridae Family, Svunavirus Genus, Hosts Geobacillus sp.)

GBSV1 was isolated from an off-shore location in shallow waters at the depth of 100 m. It was propagated in thermophilic *Geobacillus* sp. 6k51 (GenBank DQ141699), which is an aerobic rod-shaped bacterium growing at 45–85 °C [25]. It was cultivated and infected with GBSV1 at 65 °C, supplemented with Ca^2+^ and Mg^2+^ ions at pH app. 7. The 16S rDNA analysis revealed that this host shared 99.93% sequence identity with that of *G. toebii* mc-14 (GenBank EU214628). TEM analysis of GBSV1 showed a typical *Myoviridae* morphology with a 60 nm hexagonal head and 80–150 nm contractile tail. The GBSV1 genome analysis is composed of linear, 44.4% G+C dsDNA of 34,683 bp, coding for 54 putative ORFs. Thirty-three of them exhibit sequence similarities to genes from seven species of *Geobacillus* or *Bacillus* and to the bacteriophages that infect them. This may indicate their biological role of upmost importance in promoting genetic exchanges among *Bacillus* and/or *Geobacillus* bacteria: short-term adaptation, long term evolution of bacteria and population genetics. Twenty-two ORFs were bioinformatically annotated and five structural proteins of the purified GBSV1 were identified by proteomic analyses. The ORFs are grouped into functional clusters on the genome. Interestingly, seven of the GBSV1 ORFs exhibit sequence similarities with the genes from the bacteria relevant to human diseases. The authors hypothesize that thermophages may be the potential evolutionary connection between thermophiles and human pathogens. The GBSV1 shares over 90% proteome similarity with BV1, as based on genomes analysis. Two groups of genes exhibit high similarity between GBSV1, BV1 and phiOH2: (i) lysis-related proteins—a probacteriophage anti-repressor and lysin with >95% similarity, and (ii) bacteriophage genome maintenance proteins and integration—integrase, >95%, recombinase—>65% similarity [25]. These similarities indicate common, possibly interchangeable, infection modules across thermophilic host species of distinct bacterial phyla [3].

### 3.43. BV1 (Myoviridae, Family, Svunavirus Genus, Hosts Geobacillus sp.)

BV1 is highly similar to GBSV1 (see previous section) and the characteristics are essentially the same, with minor differences: a hexagonal head is 60 nm in diameter and a tail 90nm in length and 10 nm in width, the genome is 44.8% G+C, 35,055 bp. It contains 54 ORFs, classified into eight functional groups. SDS-PAGE analysis of purified BV1 revealed that it contains seven major polypeptides with molecular weights of app.: 100, 72, 42, 33, 31, 28 and 26 kDa. BLAST analysis revealed that 38 ORFs exhibited similarities to proteins present in databases. Interestingly, a diverse pattern of similarities suggests that BV1 could be involved in gene transfer between the *Bacillus* and *Geobacillus* species [58].

### 3.44. D6E (Myoviridae Family, Genus ND, Host Geobacillus sp.)

D6E was isolated from a deep-sea hydrothermal field (12°42′29″ N, 104°02′01″ W, depth of 3083 m) at the same location as GVE1, along with the same host *Geobacillus* sp. E26323 and was propagated at 65 °C. TEM results have shown that D6E is a myovirus with an icosahedral head (60 nm), a contractile tail (16 nm in width and 60 nm in length), decorated with a tail fiber (4 nm in width and 60 nm in length). The bacteriophage genome of 49,335 bp is composed of 46% G+C dsDNA, possibly circular, as based on restriction and PCR analysis. It contains 49 ORFs, with 30 of them having bioinformatically assigned homologies/function, grouped in functional clusters: (i) DNA packaging and capsid assembly, (ii) tail assembly, (iii) lysis and lysogeny, (iv) DNA replication and transcription. D6E genome does not code for any tRNA genes. Comparative genomics and proteomics analyses have indicated an extensive mosaicism of D6E genome with other mesophilic and thermophilic bacteriophages, including GVE1. The authors conclude, that there were at least three recombination events during GVE1 evolution: (i) the overall gene organization, (ii) recombination between gene boundaries and (iii) mutations in genes. As determined on SDS-PAGE and mass spectrometry 10 virion-associated proteins biosynthesis were confirmed [14].

### 3.45. ϕOH2 (phiOH2) (Family/Genus ND, Host G. kaustophilus)

Bacteriophage ϕOH2 is closely grouped with *Bacillus* virus 1 BV1 thermophage [3]. It was found in the sediment of a hot spring as lytic, infecting factor for *G. kaustophilus* NBRC102445. The genome of bacteriophage ϕOH2 is integrated into the lysogenic host genome *G. kaustophilus* GBlys, thus being a prophage. The host was cultivated at 55 °C. Its genome of 3,541,481 bp consists of 216 sequence contigs with OH2 prophage genome (38,099 bp, 45% G = C, 60 ORFs), situated astride contigs GBL0024 and GBL0110 and flanked by the host genes encoding GroES, GroEL. The host transposase gene is located next to the OH2 genome. A putative integrase gene is located at the terminus of the OH2 sequence. There are no excisionase gene homologues, and the attachment sites located next to the integrase gene are dissimilar to those coded by bacteriophages [59]. BLAST analysis show the presence of completely or partially homologous ϕOH2 sequences in many ‘*Bacillus* group’ bacteria and bacteriophages, which indicates intense horizontal gene transfer within the group.

### 3.46. GBK2 (Siphoviridae Family, Genus ND, Host G. kaustophilus)

GBK2 bacteriophage was extracted out of the compost pile using a liquid culture of *G. kaustophilus* ATCC8005, cultivated at 55 °C. GBK2 has a circularly permuted dsDNA genome of 39,078 bp and 43% G+C content. The genome contains seven copies of a 17-bp inverted DNA repeat, which forms a potential origin of replication for GBK2. Bioinformatics analysis of the genome reveals 62 putative ORFs. Twenty-five of them show homology to the known proteins and 37 are proteins with undetermined functions. The ORFs are grouped into functional clusters: (i) DNA packaging, (ii) structural proteins, (iii) lysis, (iv) unknown, (v) DNA replication/metabolism. Overall, the genome exhibits a limited DNA sequence similarity to any other sequenced bacteriophages [1]. It is intriguing that GBK2 is more closely related to the bacteriophages that infect mesophilic hosts than to those that infect thermophilic *Geobacillus* sp. or other related thermophiles species. The overall genomic arrangement of GBK2 is quite similar to that of mesophilic bacteriophage SPP1, infecting *B. subtilis* [22]. Fourteen ORFs are coding proteins that are homologous to SPP1 bacteriophage and most of them are involved in DNA replication, while two ORFs are predicted to encode capsid structural proteins. DNA-related genes, include those coding for: exonuclease/recombinase, recombinase of RecT-type, helicase, helicase loader and three DNA metabolism enzymes [18]. Furthermore, in SPP1 genome there are three genes, non-essential for growth, of unknown function. Nevertheless, homologs of these genes are found in GBK2 bacteriophage. It was concluded that they may play a significant role in the thermophage life cycles under certain conditions [60]. These functional and sequence similarities point to a common origin of SPP1 and GBK2 bacteriophages or a massive horizontal gene transfer.

### 3.47. GVE3 (Siphoviridae Family, Genus ND, Host G. thermoglucosidasius)

GVE3 bacteriophage is a B1 morphotype group *siphovirus* that infects, very specifically, the thermophilic bacteria *G. thermoglucosidasius* as the only susceptible host, propagated at 60 °C. The bacteriophage has an isometric head of 90–100 nm diameter and a non-contractile tail of 210 nm. The plaques had characteristic “bulls-eye” morphology, suggesting host lysogeny [61] with several bacterial colonies growing inside plaques. Once isolated, the bacteria from those colonies were resistant to GVE3 infection. The lysogenic host genome sequencing revealed that the GVE3 genome had inserted into the specific location, attB site, with a 23-bp sequence that was duplicated upon insertion. The bacteriophage GVE3 encodes a putative pyrimidine nucleoside phosphorylase, situated downstream of GVE3 resolvase gene. The attP site is located within the gene. Apparently, the host’s pyrimidine nucleoside phosphorylase is inactivated upon the GVE3 genome insertion.

The presence of a GVE3-encoded pyrimidine nucleoside phosphorylase suggests an obligatory requirement for its activity: upon GVE3 genome integration the host relies on the bacteriophage enzyme. There is an alternative explanation: if the enzyme is not essential for either the GVE3 or the host, since the mutation in the pyrimidine nucleoside phosphorylase-coding gene is non-lethal, this may suggest that GVE3 is a specialized transducing thermophage [62]. Interestingly, the genome size is 141,298 bp comprising the largest known among the bacteriophages that infect *Geobacillus* sp., and has a unique DNA sequence, with no close relatives. A large number of 202 putative ORFs were bioinformatically detected and, out of those, putative functions to 62 of them were assigned. As observed in a number of other members of the *Siphoviridae*, the GVE3 genome displays the functional clusters [62]. Remarkably, GVE3 resembles GBK2 in that it is apparently most closely related to the mesophilic *B. anthracis* bacteriophage vB_BanS_Tsamsa, rather than the *Geobacillus*-infecting bacteriophages. Tetranucleotide usage deviation analysis confirms this finding, indicating that the GVE3 genome sequence correlates mostly with *B. anthracis* and *B. cereus* sequences, rather than *Geobacillus* sp. sequences [62,63], which again points to widespread genetic recombination among meso- and thermophilic ‘*Bacillus* group’ bacteriophages. The analysis also suggests that there exist a different ‘natural’ host, unidentified *Geobacillus* sp., with tetranucleotide usage deviation patterns more similar to the two *Bacillus* species that might be the “natural” hosts for GVE3. Alternatively, the possibility exists that GVE3 has evolved from a mesophilic counterpart and that the correlation to mesophilic *Bacillus* species is an evolutionary link to its heritage. Similar findings have been shown for GBK2 [64]. The GVE3 genome sequence has a much lower G+C content (29.6%) than its host *G. thermoglucosidasius* (44%) [62], which is also observed for most bacteriophage–host pairs. It is suggested that its base composition bias might have resulted from a competition for metabolic resources and/or reflects an adaptation to thermophily [65]. The genome GC skew analysis has suggested a location of a replication terminus. A search for direct and inverted repeats of 7–30 bp resulted in finding as many as 582 repeats, including two invert repeats (TATTTTTT/TAATTAT) predicted to be part of the origin of the replication. The large genome segment, DNA replication, modification and repair, contains tens of genes, not all of them with assigned functions. These make the GVE3 enzymatic machinery remarkably independent, including three subunits diverging hosts DNA polymerase to the bacteriophage replication and three DNA modification methyltransferases, protecting the genome from restriction, among others [62].

### 3.48. AP45 (Siphoviridae Family, Genus ND, Host Aeribacillus sp.)

AP45 bacteriophage was isolated from a soil sample collected in the Valley of Geysers, Kamchatka, Russia. It was propagated in thermophilic *Aeribacillus* sp. strain CEMTC 656, a Gram-positive, aerobic, rod-shaped, non-motile, forming sub-terminally located endospores. The optimum growth temperature was at 50–65 °C in nutrient broth at pH 6.5, containing 0.3 M NaCl. AP45 was propagated at 55 °C. The host range is very narrow, out of 13 tested *Geobacillus* sp., only *G. icigianus* CMBI G1w1, also isolated from the Valley of Geysers, was supporting infection. AP45 exhibited high thermostability and a latent period of approximately 200 min, with a burst size of approximately 40 bacteriophage particles/infected cell. The bacteriophage produces both a ‘halo’ around clear plaque centers and ‘bulls-eye’ morphology, indicating secretion of a cell envelope depolymerase and suggesting formations of host lysogeny. The colonies isolated from the plaques proved to be resistant to AP45 infection, however, PCR using primers to AP45 has failed, thus, it is not clear whether other resistance mechanisms take place. TEM analysis of AP45 indicated *Siphoviridae* morphology with an icosahedral head of 60 nm with a diameter and a tail approximately 160 nm in length. The AP45 genome analysis has shown that it is composed of 38.3% G+C dsDNA of 51,606 bp, containing 71 ORFs, 40 of them have had putative functions predicted bioinformatically. Furthermore, ORFs were divided into three functional clusters: (i) nucleic acids metabolism and regulation of the viral life cycle; (ii) structural proteins; (iii) lysis of bacterial cells. No genes encoding DNA and RNA polymerases were identified, thus, AP45 apparently uses host polymerases. The AP45 genome exhibited limited similarity to other bacteriophage sequences. The highest value of 36% was detected for thermophilic *Geobacillus* myovirus D6E. Interestingly, the majority of putative AP45 proteins exhibited a higher similarity to proteins from bacteria belonging to the *Bacillaceae* family, than to bacteriophages. Moreover, over half of the putative AP45 ORFs were highly similar to probacteriophage sequences of the *Aeribacillus pallidus* strain 8m3. It was suggested that the AP45 bacteriophage and revealed probacteriophages might be members of a new genus within the *Siphoviridae* family [27].

### 3.49. Series of 5 Bacteriophages from Haloalkaline Lake Elmenteita of Kenyan Rift Valley (Section 3.49, Section 3.50, Section 3.51, Section 3.52, Section 3.53 and Section 3.54 Below)

All bacteriophages were isolated from Lake Elmenteita [66], situated at 0°27′ S 36°15′ E on the floor of the Kenyan Rift Valley, at 1776 m above sea level without a direct water outlet. This area has a hot, dry and semi-arid climate, which results in rapid evaporation rates. The alkalinity of the water is high alkaline with pH above 9 and with a high concentration of carbonates, chlorides and sulphates [67]. The water temperature ranges between 30 °C and 40 °C. Comparison of genome sizes, coding density, number of ORFs, GC% and gene organizations revealed that the bacteriophages had no relationship with each other as well as no significant similarities in the GenBank^®^ sequence data. Furthermore, there is evidence of genes mosaicism to described genes with conserved domains [66]. The bacterial hosts were not described in details. Nevertheless these were extremophilic bacteria with regard to salinity and alkalinity from the ‘*Bacillus* group’, possibly also entering moderate thermophilic range. No bacteriophages characteristics are available at the time of this review submission, except bacteriophages genomes bioinformatics analysis. The authors cite ongoing publication, which will contain descriptions of the bacteriophages [66].

### 3.50. vB_BpsS-36 (Family/Genus ND, Host B. pseudalcaliphilus)

The vB_BpsS-36 genome is composed of 50,485 bp dsDNA with 41.1% GC content. It codes for 68 ORFs distributed on both forward and reverse strands and six transcription terminators with coding density of 91.6%. On the DNA level, the bacteriophage shows essentially no homologies to other bacteriophages, while on the amino acids level there exists weak similarities to short regions of *Bacillus* bacteriophages phages Tsamsa, Riggi and CampHawk. A conserved replication factor was detected in the genome, suggesting that vB_BpsS-36 employs DNA polymerase III subunit alpha for DNA replication. Furthermore, vB_BpsS-36 codes for replication proteins—domains DnaB and DnaG [66]. 

### 3.51. vB_BpsM-61 (Family/Genus ND, Host B. pseudofirmus)

The vB_BpsM-61 genome is composed of 48,160 bp dsDNA with 43.5% GC content. It codes for 75 ORFs distributed on both forward and reverse strands and eight transcription terminators with coding density of 93.0%. The bacteriophage exhibits weak similarities to *Bacillus* bacteriophage PM1 and *Geobacillus* phage GBK2 in cluster coding for structural components. The vB_BpsM-61 genome is also coding for putative proteins: replicative DnaC, helix destabilizing Ssb, dUTPase, Holliday junction resolvase [66]. 

### 3.52. vB_BboS-125 (Family/Genus ND, Host B. bogoriensis)

The vB_BboS-125 genome is composed of 58,528 bp dsDNA with 48.6% GC content. It codes for 81 ORFs distributed on forward strands only and six transcription terminators with coding density of 92.2%. The bacteriophage exhibits weak similarities to other bacteriophage proteins, more pronounced to structural components and DNA packaging located in genomic regions in *Exiguobacterium* sp. AB2 and *Brevibacillus* sp. CF112, apparently being not-annotated prophage segments. In addition, the vB_BboS-125 shows similarities to genes with conserved domains of the replication cluster, helicase and primase, present in the genome of *Brevibacillus* sp. CF112. Furthermore, the vB_BboS employs a host’s DNA polymerase I for replication. Interestingly, the bacteriophage terminase did not cluster with previously described bacteriophages, but formed a distinct phyletic line, which suggests that vB_BboS-125 belongs to a new genus of bacteriophages [66].

### 3.53. vB_BcoS-136 (Family/Genus ND, Host B. cohnii)

The vB_BcoS-136 has the largest genome of an entire group of eight bacteriophages found in haloalkaline in Lake Elmenteita, 160,590 bp dsDNA with low GC content of 32.2%. It codes for 240 ORFs distributed on both forward and reverse strands, 15 transcription terminators and 17 tRNA with a coding density of 93.0%. A very high number of tRNA coding genes suggest substantial differences in codon usage between the bacteriophage and its host, thus, needed for efficient translation and vB_BcoS-136 propagation. Furthermore, it may suggest that the bacteriophage relatively recently has adopted its host. The bacteriophage exhibits very low similarities to *Bacillus* bacteriophage Tsamsa. BcoS-136 terminase is dissimilar to other bacteriophage’s terminases with an already known DNA packaging strategy, but groups in a distinct phyletic line, thus pointing to the possibility of the existence of another new genus. vB_BcoS-136 utilizes a host’s DNA polymerase III subunit alpha and codes for other DNA replication/DNA metabolism putative proteins: DNA ligase, DNA gyrase, ribonuclease HI and integrase. The latter suggests that vB_BcoS-136 can also enter lysogenic mode [66].

### 3.54. vB_BpsS-140 (Family/Genus ND, Host B. pseudalcaliphilus)

The vB_BpsS-140 genome is composed of 55,091 bp dsDNA with 39.8% GC content. It codes for 68 ORFs distributed on forward strands only and four transcription terminators with coding density of 91%. The vB_BpsS-140 encoded genes for proteins similar to homologs of a terminase and some structural proteins of *Bacillus* bacteriophages IEBH and 250, respectively. Homology comparison of the large terminase subunit shows that vB_BpsS-140 clusters with P22 bacteriophage, which is established as a model bacteriophage that follows headful packaging strategy [68]. As this protein is considered the most universally conserved gene sequence among bacteriophages [69], it is used to get insight into evolutionary relationships among distant bacteriophages [70]. Interestingly, the bacteriophage codes for atypical endopeptidase endolysin, are absent in other bacteriophages published by the authors [66].

## 4. Scientific, Biotechnology, Environmental and Medical Potential—A Short Note

In general, bacteriophages, and their corresponding enzymatic activities, offer great potential for the development of agents for the control of human pathogens [71]. Bacteriophage bionanostructures are used in science, industry and medicine, as exemplified by bioimaging diagnostics, ultrasensitive biomarker detection, targeted drug and gene delivery, improved tissue formation, directed stem cell differentiation, new generation vaccines and nanotherapeutics for targeted disease treatment, among others [71]. These applications may be substantially improved if based on robust, thermostable bacteriophage bionanoparticles. The study of *Geobacillus* host bacteria, their bacteriophages and their mutual interaction, presents great potential for gaining knowledge about the functioning of high temperature ecosystems, including the thermophages crucial role as regulators of bacterial populations. No less importantly, this knowledge is likely to help improve the known processes or to develop new processes in biotechnology. The list of viral genome sequences reported thus far in the National Center for Biotechnology Information Genome database, as of June 2021, includes 10,607 genomes; 3746 of them being bacterial hosts, with only four hosts listed under ‘*Geobacillus*’. This proportion shows that there are significant gaps in the understanding of the genetics of the ‘*Bacillus* group’ thermophages as well as of the type and dynamics of their biological processes at a local and global scale. 

Even though thermophiles and extreme thermophiles are presently attracting substantial attention, the area remains in large part a ‘terra incognita’ in terms of approaching understanding of mesophilic host-bacteriophage systems (such as *E. coli*/bacteriophage lambda). Nevertheless, they can provide model systems for deciphering the molecular biology and biochemistry of adaptations towards life at high temperatures and their effects on atypical ecosystems, and can affect many biogeochemical and ecological processes.

The scientific interest in thermophilic bacilli, especially *Geobacillus* sp., is fully justified, due to their potential applications in biotechnology and environment remediation processes. Nevertheless, thermophilic bacteriophages can cause huge economical losses, upon undesired infections of industrial fermentations. Bacteriophage-sensitive processes are shown in the following examples.

As *Geobacillus* sp. possesses the ubiquitous capability to metabolize various organic compounds, including hydrocarbons, the fermentation of C-5 and C-6 sugars is being used to biosynthesize ethanol for biofuel and organic acids production [72]. Even very ‘difficult’ substrates, such as cellulose, the most abundant organic carbon reservoir on Earth, are effectively degraded by *Geobacillus* sp. cellulases.

More thermostable enzymes, coded by *Geobacillus* sp., are also of interest to the biotechnology industry, such as: proteases, amylases, polysaccharides, lipases, glycanases and herbicides metabolizing enzymes for agricultural biotechnology [10,73,74,75,76].

Certain *Geobacillus* sp. are also capable of the destruction of quorum sensing in some Gram-negative bacteria [10]. Furthermore, some strains of *G. thermoleovorans* are secreting large bacteriocins that cause lysis of various other bacteria, including pathogenic *Salmonella typhimurium* [77]. These capabilities could be a basis for future therapeutic methods. Thus, cataloguing bacteriophages and studying their interactions with their hosts is also important for practical purposes. Furthermore, bacteriophages can affect the human microbiome, including facultatively thermophilic ‘*Bacillus* group’ species. Several clades from genus *Bacillus* of the *Bacillaceae* family involve bio-safe species, regarded as bimodal probiotic microbiobiota of humans and animals [78]. These are strains generally derived from environmental locations such as soil, marsh and plants, but are also found in animal’s and human’s skin, gastrointestinal tract or respiratory system. Several *Bacillus* species are typically encountered in traditional fermented foods, especially those of Asian or African origin that are prepared prevalently from protein rich soybeans or locust beans. The outstanding potential for the survival of environmental probiotics results from their ability to form durable spores [79] capable of enduring harsh conditions of draught, UV exposure, extreme temperatures and pH values, the presence of organic and inorganic chemicals, salts, detergents and heavy metal ions. During the past decade, probiotic *Bacillus* species, including *B. subtilis*, *B. coagulans*, *B. clausii* and *B. licheniformis*, are of growing interest and seem to be promising models for biotechnological applications, involving industrial, agricultural, medical or, typically, scientific applications [80,81]. Pathogenic activity cases of ‘*Bacillus* group’ are extremely rare [82], thus, no phage therapy against these bacteria has been developed for human use. However, for animal protection purposes, successful phage therapy has been used, employing FBL1 bacteriophage preparation for combating the mortality of the white Pacific shrimp, *Litopenaeus vannamei*, infected by *B. licheniformis*. Some strains of these bacteria are capable of extending their upper temperature growth limit up to approximately 50 °C, thus stepping into the thermophilic range. The FBL1 not only impaired its host growth, but also prevented the formation of biofilm [83]. Besides biofilm degradation/prevention, there are other molecular mechanisms that can be exploited to control bacterial population. The bacteriophage SPO1 of *B. subtilis* is coding for Gp44 protein, which is part of an unusual molecular mimicry strategy inhibiting host RNA polymerase. Gp44 is composed of a DNA binding motif, a flexible DNA mimic domain and a random-coiled domain. Such composition allows selective binding to bacterial RNA polymerase through β and β’ subunits, blocking bacterial growth. This a non-specific mechanism that SPO1 employs to target different bacterial transcription apparatus, regardless of the structural variations of RNA polymerases. This opens up the possibility for the generation of genetically modified SPO1 for the purpose of targeted gene therapy [84,85].

It is known that these industrial and medical biotechnology applications can be dramatically affected by undesired thermophage infections. On the other hand, thermophages are important in biotechnology, not only because of their potential to cause big economic losses but also because they also code for a number of valuable enzymes, which are often more robust than those of their hosts due to the rapid nature of a high temperature thermophage infection requirements. Some of these enzymes, with a big potential for novel applications, are the less recognized endolysins-lysozymes and depolymerases-glycosidases of thermophage origin [1,86,87]. These enzymes could be used for treating industrial installations overgrown with various bacteria as well as in biofilm removal for medical usage [1,86,87]. Since the critical initial step in a bacteriophage infection is the adsorption to its compatible receptor on the surface of the bacterial cell, the presence of exopolysaccharide envelopes can effectively block this stage. Some bacteriophages are producing a ‘halo’ around clear plaque centers, indicating a diffusing factor, hydrolyzing and allowing for the penetration of the extracellular exopolysaccharide matrix—a major bacterial biofilm component [88,89,90]. A number of thermophages produce thermostable depolymerases-glycosylases, endolysins (lysozymes) and holins, involved in actions against external bacterial layers, capsule, cell wall, cell membrane, protecting the internal cell cytoplasmatic environment [64]. The thermophage TP-84 codes for all three types of enzymes [2]. The combination of three such types of thermostable, robust enzymes in a cocktail for biofilms removal may become a powerful tool in industrial, environmental and medical applications. The ability of bacteria to form biofilms is an important element of their pathogenicity, and biofilm destruction is of high interest in medicine as a potential alternative or supplement to existing therapies [91]. TP-84 is a bacteriophage that deprives *G. stearothermophilus* bacteria of their capsule, thus facilitating the permeation of bacteriophages into deeper biofilm layers and lysis of the susceptible bacterial cells. Considering the rather broad specificity of TP-84 lytic enzymes, if applied to pathogen biofilms, it may prevent their formation and contribute to eradication of the biofilm bacteria [91]. Furthermore, thermophages often produce other useful enzymes, for example, GVE3 is encoding DNA ligase, DNA polymerase, among others [92]. Additionally, there are works being conducted to develop a GVE3 thermophage-based system for the introduction of novel or engineered metabolic and biosynthetic pathways [61]. Some of the lysogenic thermophages contain integrases, which are of growing importance for the genetic engineering of living eukaryotic cells, as they can mediate precise site-specific recombination between two different sequences [93,94]. An application unexplored thus far would be the construction of a robust, thermostable phage display system for directed evolution *in vitro*. Such systems have made a major breakthrough in molecular biology and biotechnology, so that the inventors were honored with the 2018 Nobel Prize.

## Figures and Tables

**Table 1:** New (this work) experimentally confirmed functional CDSs of bacteriophage TP-84 genome—an update 1.

CDS Name	CDS Length (bp)	Location in the Genome (bp)	CDS Arbitrary Orientation	Polypeptide Length (aa)	Predicted Polypeptide Molecular Weight (kDa)	Experimentally Determined Polypeptide Molecular Weight (kDa)	Predicted Isoelectric Point	Hypothetical Function (Analysis)	Confirmed by Proteomic Analysis
TP84_01	567	13–579	+	188	22.4	22.4	7.76	terminase, small subunit	terminase, small subunit
TP84_02	1299	576–1874	+	432	50.0	50.1	7.74	terminase, large subunit	terminase, large subunit
TP84_04	354	2586–2939	+	117	13.6	ND	4.36	unknown	unknown
TP84_15	324	10358–10681	+	107	11.9	ND	6.26	unknown	unknown
TP84_20	375	12687–13061	+	124	14.7	14.7	9.80	unknown	unknown
TP84_26	2976	20179–23154	+	991	112.2	112.9	5.49	glycosylase	glycosylase
TP84_27	432	23240–23671	+	143	15.5	15.5	6.20	holin	holin
TP84_30	573	25329–25901	+	190	21.7	21.7	4.98	unknown	unknown
TP84_33	291	26597–26887	+	96	11.0	ND	5.10	unknown	unknown
TP84_41	273	28824–29096	+	90	10.6	ND	9.69	unknown	unknown
TP84_43	309	29330–29639	+	102	11.0	ND	9.81	unknown	unknown
TP84_48	2265	30527–32791	+	754	86	ND	7.09	unknown	unknown
TP84_49	474	32871–33344	+	157	18.0	ND	5.85	unknown	unknown
TP84_58	318	37942–38259	+	105	12.3	ND	4.92	replicative helicase inhibitor	replicative helicase inhibitor
TP84_59	1305	38259–39563	+	434	49.1	49.2	5.53	replicative DNA helicase	replicative DNA helicase
TP84_60	672	39632–40303	+	223	26.4	26.4	8.41	unknown	unknown
TP84_61	435	40281–40715	+	144	16.7	ND	9.08	HNH homing endonuclease	HNH homing endonuclease
TP84_70	330	43704–44033	+	109	12.7	ND	9.47	unknown	unknown
TP84_73	264	44746–45009	+	87	10.4	ND	5.60	unknown	unknown

**Table 2:** Classification and major data describing ‘*Bacillus* group’ thermophilic bacteriophages (chronologically as discovered).

N°	Bacteriophage Species	Virus Family, Genus	GenBank Accession Number	Host (Used for Propagation)	Genome			Original Discovery Reference	Isolate Location	Life Cycle	Growth Temperature (°C, Optimal/Range) and pH (Optimal/Range)
					Type and Size [bp]	ORFs	G+C [%]				
1	‘thermophilic lytic principle’	ND	ND	*B. stearothermophilus* T60	ND	ND	ND	Koser, 1926	sewage polluted river water (USA)	lytic	52–60, app. 7
2	‘thermophilic bacteriophage’	ND	ND	ND	ND	ND	ND	Adant, 1928	ND	lytic	52–55
3	‘thermophilic bacteriophage’	ND	ND	*thermophilic bacterium* no. 10	ND	ND	ND	White et al., 1954, 1955	greenhouse soil (USA)	lytic	65 (50–70), 7
4	‘thermophilic bacteriophage’	ND	ND	*Bacillus* sp.	DNA, RNA(?)	ND	ND	Onodera; 1959; Onodera 1961	compost (Japan)	lytic	65 (55–70), 7.2
5	TP-84	*Siphoviridae,* *Tp84virus*	KY565347.1	*B. stearothermophilus* strain 10	circular, dsDNA, 47,718	81	54.5	Saunders & Campbell, 1964	greenhouse soil (USA)	lytic	58 (43–76), 7.2
6	φμ-4	ND	ND	*B. stearothermophilus*	ND	ND	ND	Shafia & Thompson, 1964	ND	lytic/lysogenic	50–65, 7
7	TP-1	*Siphoviridae* (putative)	ND	*B. stearothermophilus*	dsDNA, app. 18,516 bp, (MW 12.1 Mda)	ND	42	Welker and Campbell, 1965	ND	lysogenic/lytic	55 (50–65), 7
8	ST1	*Siphoviridae* *Myoviridae* (putative)	ND	*B. stearothermophilus* strain S13	dsDNA	ND	43	Carnevali & Donelli, 1968	ND	lytic	60, app. 7
9	Tφ3	*Siphoviridae*	ND	*B. stearothermophilus* ATCC 8005 S^R^	ds DNA, app. 35,700 (MW app. 23.2 MDa)	ND	40.2	Egbert&Mitchel, 1967; Egbert, 1969	soil (USA)	lytic	60, 7.3
10	GH5	ND	ND	*B. stearothermophilus* NCA1518	ND	ND	ND	Humbert & Fields, 1972	greenhouse soil (USA)	lytic	42.5–67, app. 7
11	GH8	*Siphoviridae*	ND	*B.stearothermophilus* NCA1518	ND	ND	ND	Humbert & Fields, 1972	greenhouse soli (USA)	lytic	42.5–67, app. 7
12	PhB1	*Siphoviridae*	ND	*Bacillus* sp. strain B	ND	ND	ND	Junger & Edebo, 1972	farm soil (Sweden)	lytic	55, 7.3
13	D5	ND	ND	*B. stearothermophilus* NRS T91, ATCC7953	ND	ND	ND	Reanney & Marsch, 1973	ND	lytic	45 (30–55), app. 7
14	D6	ND	ND	*B. stearothermophilus* NRS T91, ATCC7953	ND	ND	ND	Reanney & Marsch, 1973	ND	lytic	45 (30–55), app. 7
15	D7	ND	ND	*B. stearothermophilus* NRS T91, ATCC7953	ND	ND	ND	Reanney & Marsch, 1973	ND	lytic	45 (30–55), app. 7
16	D8	ND	ND	*B. stearothermophilus* NRS T91, ATCC7953	ND	ND	ND	Reanney & Marsch, 1973	ND	lytic	45 (30–55), app. 7
17	φNS11	*Sphaerolipoviridae*, (putative)	ND	*B.acidocaldarius* TA6	dsDNA	ND	ND	Sakaki & Oshima, 1976	hot spring (Beppu, Japan)	lytic	60, 3.5 (2–5)
18	JS001	ND	ND	*B. caldotenax*	dsDNA	ND	ND	Sharp et al., 1986	ND	lytic/lysogenic	55 (50–70), 7.3 ± 0.2
19	JS004	ND	ND	*Bacillus thermophile* RS 239	dsDNA	ND	ND	Sharp et al., 1986	silage	lytic	55 (50–70), 7.3 ± 0.2
20	JS005	ND	ND	*B. thermophile* RS 239	dsDNA	ND	ND	Sharp et al., 1986	rotting straw	lytic	55 (50–70), 7.3 ± 0.2
21	JS006	ND	ND	*Bacillus thermophile* RS 239	dsDNA	ND	ND	Sharp et al., 1986	compost	lytic	55 (50–70), 7.3 ± 0.2
22	JS007	ND	ND	*Bacillus thermophile* RS 240	dsDNA	ND	ND	Sharp et al., 1986	silage	lytic	55 (50–70), 7.3 ± 0.2
23	JS008	ND	ND	*Bacillus thermophile* RS 241	dsDNA	ND	ND	Sharp et al., 1986	rotting straw	lytic	55 (50–70), 7.3 ± 0.2
24	JS009	ND	ND	*Bacillus thermophile* RS 242	dsDNA	ND	ND	Sharp et al., 1986	stable manure	lytic	55 (50–70), 7.3 ± 0.2
25	JS010	ND	ND	*Bacillus thermophile* RS 242	dsDNA	ND	ND	Sharp et al., 1986	compost	lytic	55 (50–70), 7.3 ± 0.2
26	JS011	unclassified family	ND	*Bacillus thermophile* RS 239	dsDNA	ND	ND	Sharp et al., 1986	silage	lytic	55 (50–70), 7.3 ± 0.2
27	JS012	ND	ND	*Bacillus thermophile* RS 239	dsDNA	ND	ND	Sharp et al., 1986	compost	lytic	55 (50–70), 7.3 ± 0.2
28	JS013	ND	ND	*B.stearothermophilus* NCA 1503	dsDNA	ND	ND	Sharp et al., 1986	soil	lytic	55 (50–70), 7.3 ± 0.2
29	JS014	ND	ND	*B.stearothermophilus* NCA 1503	dsDNA	ND	ND	Sharp et al., 1986	rotting straw	lytic	55 (50–70), 7.3 ± 0.2
30	JS015	ND	ND	*B.stearothermophilus* NCA 1503	dsDNA	ND	ND	Sharp et al., 1986	compost	lytic	55 (50–70), 7.3 ± 0.2
31	JS017	ND	ND	*B. caldotenax*	dsDNA	ND	ND	Sharp et al., 1986	compost	lytic	55 (50–70), 7.3 ± 0.2
32	JS018	ND	ND	*B. caldotenax*	dsDNA	ND	ND	Sharp et al., 1986	rotting vegetation	lytic	55 (50–70), 7.3 ± 0.2
33	JS019	ND	ND	*B. caldotenax*	dsDNA	ND	ND	Sharp et al., 1986	rotting vegetation	lytic	55 (50–70), 7.3 ± 0.2
34	JS020	ND	ND	*B.s caldotenax*	dsDNA	ND	ND	Sharp et al., 1986	rotting vegetation	lytic	55 (50–70), 7.3 ± 0.2
35	JS021	ND	ND	*B. caldotenax*	dsDNA	ND	ND	Sharp et al., 1986	rotting vegetation	lytic	55 (50–70), 7.3 ± 0.2
36	JS022	ND	ND	*B. caldotenax*	dsDNA	ND	ND	Sharp et al., 1986	compost	lytic	55 (50–70), 7.3 ± 0.2
37	JS023	ND	ND	*B. caldotenax*	dsDNA	ND	ND	Sharp et al., 1986	compost	lytic	55 (50–70), 7.3 ± 0.2
38	JS024	ND	ND	*B. caldotenax*	dsDNA	ND	ND	Sharp et al., 1986	compost	lytic	55 (50–70), 7.3 ± 0.2
39	JS025	ND	ND	*B. caldotenax*	dsDNA	ND	ND	Sharp et al., 1986	compost	lytic	55 (50–70), 7.3 ± 0.2
40	JS026	ND	ND	*B. caldotenax*	dsDNA	ND	ND	Sharp et al., 1986	compost	lytic	55 (50–70), 7.3 ± 0.2
41	JS027	ND	ND	*Bacillus thermophile* RS 241	dsDNA	ND	ND	Sharp et al., 1986	compost	lytic	55 (50–70), 7.3 ± 0.2
42	BVW1 (W1)	*Siphoviridae*	ND	*Bacillus* sp. w13	dsDNA, app. 18 kb	ND	ND	Liu et al., 2006	deep-sea hydrothermal fields (West Pacific)	lytic	60, 7.0
43	GVE1 (E1)	*Siphoviridae*	ND	*Geobacillus* sp. E 26323	dsDNA, app. 41 kb	ND	ND	Liu et al., 2006	deep-sea hydrothermal fields (East Pacific)	lytic	60, 7.0
44	GVE2 (E2)	*Siphoviridae,* unclassified *Siphoviridae*	NC_009552 DQ453159	*Geobacillus* sp. E 263	linear, dsDNA, 40,863	62	44.8	Liu & Zhang, 2008a,b	deep sea (China)	lysogenic	65, 7.0
45	GBSV1	*Myoviridae,* *Svunavirus*		*Geobacillus* sp. 6k512	linear, dsDNA, 34,683	54	44.4	Liu et al., 2009, 2010	off shore hot spring, (Xiamen, China)	lytic	65, 7.2
46	BV1	*Myoviridae* *,Svunavirus*	NC_009737.2, DQ840344	*Geobacillus* sp. 6k512	linear, dsDNA, 35,055	54	44.4	Liu et al., 2009, 2010	off shore hot spring, (Xiamen, China)	lytic	65, 7.2
47	D6E	*Myoviridae*	NC_019544	*Geobacillus* sp. E 26323	circular, dsDNA, 49,335	49	46	Wang & Zhang, 2010	deep-sea hydrothermal fields (East Pacific)	lytic	65, 7.0
48	ϕOH2 (phiOH2)	*Siphoviridae*	AB823818, NC_021784	*G. kaustophilus* GBlys, *G. kaustophilus* NBRC 102445(T), lysogenic *G. kaustophilus* GBlys)	dsDNA, 38,099	60	45	Doi et al., 2013	hot spring sediment (Japan)	lytic/lysogenic	55
49	GBK2	*Siphoviridae*	KJ159566	*G. kaustophilus*	Circularly permuted, dsDNA, 39,078	62	43	Marks & Hamilton, 2014	compost (Cary, NC, USA)	lytic	55, 7.3
50	GVE3 (E3)	*Siphoviridae*	NC_029073, KP144388	*G. thermoglucosidasius*	dsDNA 141,298	202	29.6	Van Zyl et al., 2015	ND	lytic/lysogenic	60, 7.3
51	AP45	*Siphoviridae*	KX965989	*Aeribacillus* sp. CEMTC656	dsDNA 51,606	71	38.3	Morozowa et al., 2019	soil (Valley of Geysers, Kamchatka, Russia)	lytic/lysogenic	55, 7.5
52	vB_Bps-36	ND	MH884513	*B. pseudalcaliphilus*	dsDNA 50,485	68	41.1	Akhwale et al., 2019	Lake Elmenteita, (Kenya)	lytic/?	30–40< 9<
53	vB_BpsM-61	ND	MH884514	*B. pseudofirmus*	dsDNA 48,160	75	43.5	Akhwale et al., 2019	Lake Elmenteita, (Kenya)	lytic/?	30–40< 9<
54	vB_BboS-125	ND	MH884509	*B. bogoriensis*	dsDNA 58,528	81	48.6	Akhwale et al., 2019	Lake Elmenteita, (Kenya)	lytic/?	30–40< 9<
55	vB_BcoS-136	ND	MH884508	*B. cohnii*	dsDNA 160,590	240	32.2	Akhwale et al., 2019	Lake Elmenteita, (Kenya)	lytic/?	30–40< 9<
56	vB_BpsS-140	ND	MH884512	*B. pseudalcaliphilus*	dsDNA 55,091	68	39.8	Akhwale et al., 2019	Lake Elmenteita, (Kenya)	lytic/?	30–40< 9<

## Data Availability

Brief data presented in the Table 1 will be published in details elsewhere.
